# Esophagogastric Fistula: The Consequence of High-Powered Magnets Ingestion

**DOI:** 10.1097/PG9.0000000000000385

**Published:** 2023-11-13

**Authors:** Hugo Quezada, Anne E. Levine, Matthew Dellinger, Samuel Rice-Townsend, Hengqi Betty Zheng

**Affiliations:** From the * Department of Pediatrics, University of Washington School of Medicine, Seattle, WA; † Department of Pediatrics, Seattle Children’s Hospital, Seattle, WA; ‡ Division of Gastroenterology and Hepatology, Seattle Children’s Hospital, Seattle, WA; § Department of Surgery, University of Washington, Seattle, WA; ∥ Division of Pediatric General Surgery, Seattle Children’s Hospital, Seattle, WA.

**Keywords:** Fistula creation, High-powered magnet ingestion

## Abstract

A 17-month-old female had an unwitnessed ingestion of 26 high-powered magnets, resulting in the creation of an esophagogastric fistula via the left crus of the diaphragm. This case highlights a rare injury to the stomach and esophagus caused by high-powered magnets requiring surgical intervention. Furthermore, this case report illustrates the risks that high-powered magnets pose to young children. Additionally, this case highlights the importance of maintaining a high level of suspicion for ingestion in young patients along with a multidisciplinary team to manage sequelae of injury.

## INTRODUCTION

A previously healthy 17-month-old female presented with episodes concerning melena to the emergency department of a community hospital. Further history revealed that she had had 3 days of decreased oral intake before passing dark-colored stool. Parents denied any sick contacts and did not witness nor suspect any ingestions.

## CASE REPORT

**FIGURE 1.**
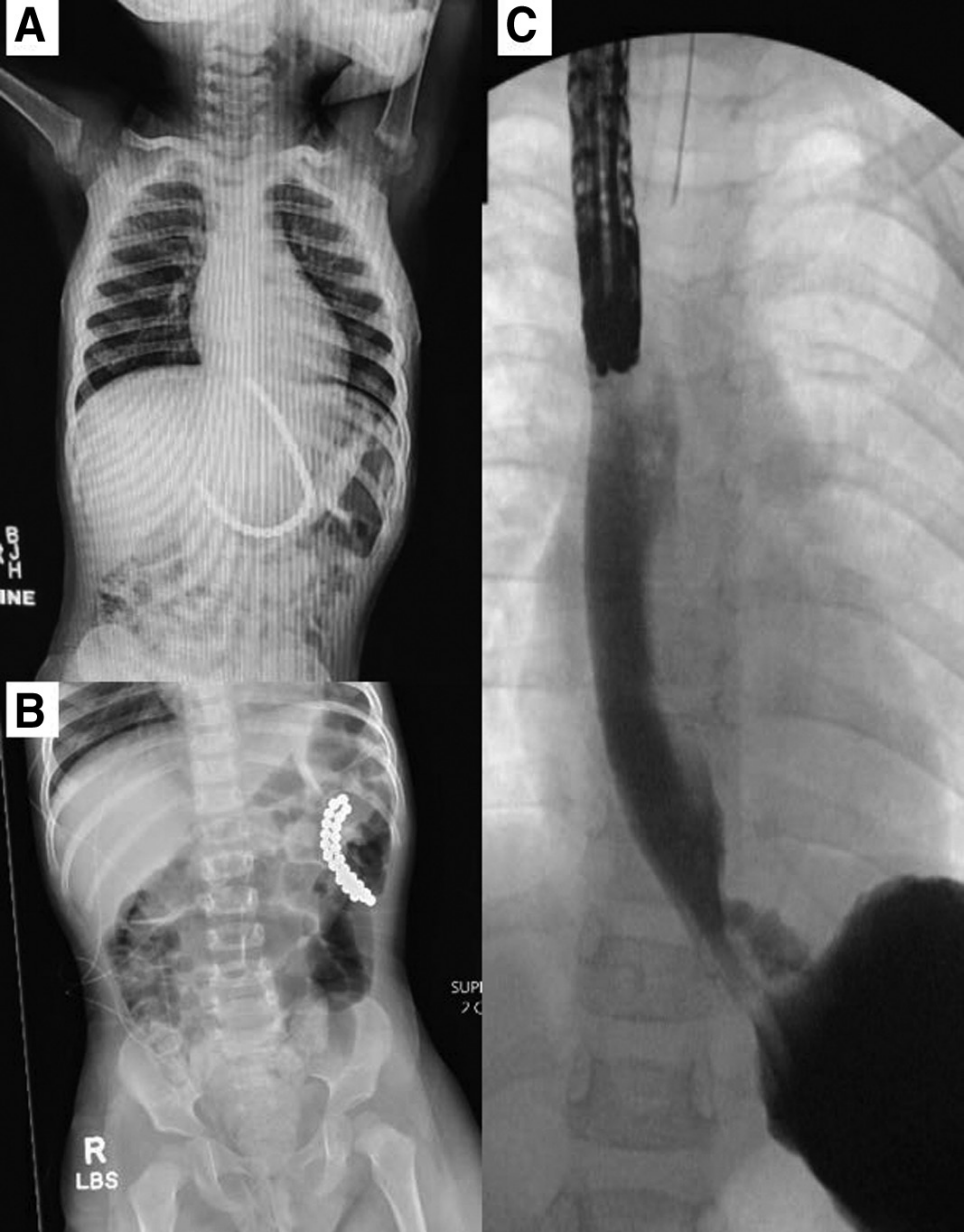
Radiographic and contrast studies showing (A) magnets lining the esophagus to the duodenum, (B) magnets looped in the stomach and (C) esophagogastic fistula created.

**FIGURE 2.**
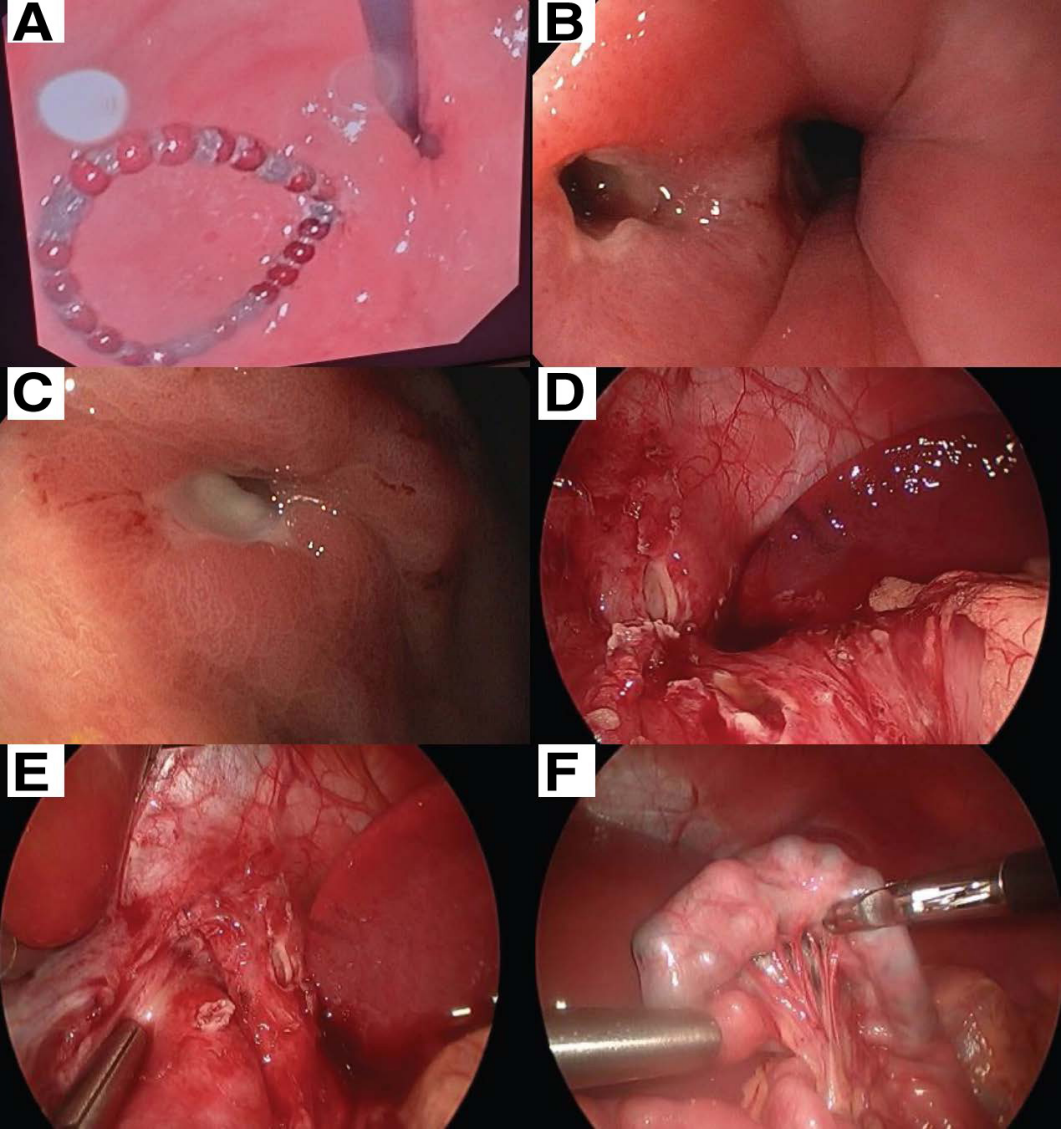
Endoscopic and laparoscopic images. A) Magnets looped in the stomach. B) Repeat endoscopy shows the tear in the esophagus. C) Corresponding perforation seen in the stomach. D) Erosions of the left crus and stomach. E) Esophageal and crus perforations. F) Magnets found at the distal jejunum.

Abdominal radiographs revealed over 20 radio-opaque round foreign bodies highly suggestive of magnets distributed from the esophagus to the duodenum (Fig. 1A). The patient was hemodynamically stable at the time of evaluation with no emesis or abdominal pain. Endoscopic removal was attempted by an adult gastroenterologist (Fig. 2A). After a single magnet was removed from the esophagus, a full-thickness underlying erosion was revealed. The decision was made to abort the procedure to remove any further magnets and to transfer to a tertiary care pediatric center. A repeat radiograph showed migration of all the magnets into the stomach and no recognizable perforation (Fig. 1B). Subsequent endoscopy was undertaken with the pediatric surgery team on standby. The esophageal and gastric erosions were again visualized, but the magnets were not in the stomach nor the proximal duodenum (Fig. 2B, C). A contrast fluoroscopic study demonstrated a fistula from the esophagus to the stomach via the left crus of the diaphragm with no extravasation of the contrast (Fig. 1C). The surgery team then proceeded with laparoscopic mobilization of the stomach to primarily suture and repair the full-thickness injuries to the esophagus and the stomach separately. The repair was reinforced with the creation of a Dor fundoplication (Fig. 2D, E). The magnets were eventually found in the distal jejunum, and 26 magnets were extracted via a small periumbilical laparotomy (Fig. 2F). Postoperatively, the patient’s diet was gradually advanced and 2 follow-up esophagrams showed a stable repair before discharge. The patient was discharged after 5 days. The family recognized the high-powered magnets as belonging to an older sibling who had used them for a science project but was unaware of how or when the patient ingested them. An outpatient esophagram performed 4 weeks postoperatively showed no leaks, and only mild narrowing at the distal gastroesophageal junction without dysphagia. Two years after her repair, she has been seen in follow-up and has no symptoms or sequelae from her injuries or repair.

## DISCUSSION

Foreign body ingestions, particularly high-powered magnets, pose a significant threat to children of all ages. They are fun, small, and colorful, but as demonstrated in this case, they can cause significant gastrointestinal injuries, including perforation and fistula formation. Previous public health information and studies have highlighted the rise in these cases particularly in children under the age of 5, with an average annual battery-related ED visit rate of 4.6 visits per 100,000 children (1). Additional studies have shown the dire consequences including the need for extensive surgical procedures and complications (2).

Over the past decade or more, both the North American Society for Pediatric Gastroenterology, Hepatology, and Nutrition and the American Academy of Pediatrics have led advocacy in patient safety measures to ban high-powered magnets (3,4). The United States Consumer Product Safety Commission first brought warnings about these magnets to light in 2007 (3–5). Advocacy with calls to lawmakers led to a brief ban on limitations in magnet size and strength from 2014 to 2016. However, this ban was lifted in 2016, and high-powered magnets returned to the market (3,4,6). Shaul et al (4) described the increased incidence of magnet ingestion at a large tertiary care children’s hospital during a postban period of 2017–2020, highlighting the continued danger of having these magnets on the market. The National Electronic Injury Surveillance System in the United States focused on these two time periods of ban on sales and postban and found a 32% increase in the number of cases of ingestions with 3,709 cases involving small/round magnets and 6,100 multiple magnets (7,8). A more recent publication looked at data from the National Poison Data System from 2008 to 2019 in the United States and found that magnet exposure increased by over 4-fold since 2018 with an increased number of hospitalizations since 2018 (9).

Over the last several years, the North American Society for Pediatric Gastroenterology, Hepatology, and Nutrition and the American Academy of Pediatrics continued to be active in regulatory debates and court appearances which led to new regulations issued by the Consumer Product Safety Commission) in September 2022 to ensure the safety of children and these magnets. Magnets must now “be either too large to swallow or weak enough to reduce the risk of internal injuries when swallowed” (10). Although this is a victory for pediatric advocates, the risks remain present as there are already millions of high-powered magnets in homes and classrooms across the United States, and these magnets are still easily available online. This risk is not unique to the United States, as several publications support the harm of high-powered magnets in Japan and the United Kingdom (11,12).

It is important to recognize that the presenting signs and symptoms of foreign body ingestions, including magnets, are broad and subtle. Thus, there should be a low threshold to include this possibility in the differential diagnosis, particularly when caring for patients who may not be able to provide accurate histories. Prompt identification is crucial to intervene endoscopically before a significant injury occurs or surgical intervention is needed. Previous cases and reports have described many types of fistulas caused by magnets but esophagogastric fistulas appear to be rare. With this case report, we hope to garner greater awareness of this potential consequence (13).

This case reinforces several key concepts regarding high-powered magnet ingestions. Radiographs are useful to determine the approximate location of the magnets, but endoscopic, direct visualization is required for confirmation. Contrast studies can elucidate damage that the endoscope cannot reveal, such as connecting fistulas and their stability. These investigations can help determine if surgical intervention is needed. Finally, a collaborative approach involving pediatric gastroenterologists and pediatric surgeons is needed to provide optimal care to children after high-power magnet ingestions (14).

## ACKNOWLEDGMENTS

Informed consent from the parent/legal guardian was obtained for this report.
